# No Association of Coffee Consumption with Gastric Ulcer, Duodenal Ulcer, Reflux Esophagitis, and Non-Erosive Reflux Disease: A Cross-Sectional Study of 8,013 Healthy Subjects in Japan

**DOI:** 10.1371/journal.pone.0065996

**Published:** 2013-06-12

**Authors:** Takeshi Shimamoto, Nobutake Yamamichi, Shinya Kodashima, Yu Takahashi, Mitsuhiro Fujishiro, Masashi Oka, Toru Mitsushima, Kazuhiko Koike

**Affiliations:** 1 Kameda Medical Center Makuhari, Chiba, Japan; 2 Department of Gastroenterology, Graduate School of Medicine, The University of Tokyo, Tokyo, Japan; 3 Department of Gastroenterology and Hepatology, Faculty of Medicine, Saitama Medical University, Saitama, Japan; The University of Hong Kong, China

## Abstract

Probably due to caffeine-induced gastric acid secretion, negative effects of coffee upon various upper-gastrointestinal diseases have been precariously accepted, despite the inadequate epidemiological evidence. Our aim is to evaluate the effect of coffee consumption on four major acid-related diseases: gastric ulcer (GU), duodenal ulcer (DU), reflux esophagitis (RE), and non-erosive reflux disease (NERD) based on the large-scale multivariate analysis. Of the 9,517 healthy adults, GU, DU, and RE were diagnosed by endoscopy, and NERD was diagnosed by the symptoms of heartburn and regurgitation without esophageal erosion. Associations between coffee consumption and the four disorders were evaluated, together with age, gender, body mass index (BMI), *Helicobacter pylori (HP)* infection status, pepsinogen I/II ratio, smoking, and alcohol. We further performed meta-analysis using the random effects model to redefine the relationship between coffee intake and peptic ulcer disease. The eligible 8,013 study subjects comprised of 5,451 coffee drinkers and 2,562 non-coffee drinkers. By univariate analysis, age, BMI, pepsinogen I/II ratio, smoking, and alcohol showed significant associations with coffee consumption. By multiple logistic regression analysis, positively correlated factors with significance were *HP* infection, current smoking, BMI, and pepsinogen I/II ratio for GU; *HP* infection, pepsinogen I/II ratio, and current smoking for DU; *HP* non-infection, male, BMI, pepsinogen I/II ratio, smoking, age, and alcohol for RE; younger age, smoking, and female for NERD. The meta-analyses could detect any association of coffee consumption with neither GU nor DU. In conclusion, there are no significant relationship between coffee consumption and the four major acid-related upper gastrointestinal disorders.

## Introduction

Coffee is one of the most widely consumed beverages in the world; in particular, Japan is one of the biggest coffee markets in Asia [1]. Coffee consumption has been reported to be associated with several diseases including peptic ulcer (PU) and gastroesophageal reflux disease (GERD), both of which are very common esophago-gastro-duodenal disorders worldwide [2]. PU is comprised of gastric ulcer (GU) and duodenal ulcer (DU), and GERD is comprised of reflux esophagitis (RE) and non-erosive reflux disease (NERD); these four are the most frequent upper gastrointestinal disorders considered to be acid-related [3]. It is generally thought that coffee intake should influence on these disorders probably due to gastric acid secretion induced by coffee containing caffeine [4]. However, results of many previous reports were still controversial: some studies denoted that PU has no association with coffee consumption [5]–[15], other studies reported the correlation between PU and coffee intake [9], [16]. For GERD, non-epidemiological studies have reported that coffee causes a relaxation of the lower esophageal sphincter [17], [18], which could increase the risk of both RE and NERD. Two epidemiological studies also implied that coffee consumption might affect the risk of GERD [19], [20], but the numbers of studies investigating the relation of coffee with GERD are at present very small. Totally, the effects of coffee consumption upon these four upper gastrointestinal disorders are still disputable matters.

To evaluate the effect of coffee consumption on four upper gastrointestinal disorders precisely, effects of many causative factors such as *Helicobacter pylori* (*HP*) infection, obesity, smoking, alcohol drinking, etc. should be taken into consideration [21]–[29]. Among the most important is thought to be *HP* infection, which is an evident risk factor for peptic ulcer diseases [30], and also an apparent preventive marker for reflux esophagitis [31]. From the standpoint of confounding variables, effects of coffee consumption upon the four upper gastrointestinal disorders should be carefully evaluated, as some reports denoted that coffee intake presents considerable association with *HP* infection, obesity, smoking, or alcohol drinking [32]–[34]. As the subjects of our present study mostly composed of Japanese, who are known to be very high prevalence of *HP* infection [35] and also known to be considerably high rate of smokers [36], a detailed investigation considering the effects of these confounding factors should be conducted.

## Materials and Methods

### Study Population

Study participants were 9,517 adults who received a medical checkup at Kameda Medical Center Makuhari from October 2010 to September 2011. In this study, all the participants were asked to respond to the Frequency Scale for the Symptoms of GERD (FSSG) [37] and also respond to the detailed questionnaire below-mentioned. They also underwent a variety of examinations such as upper gastrointestinal endoscopy, abdominal ultrasonography, blood chemistry tests, chest X-ray, physical examinations, and so on. The gender breakdown of participants was 5,675 men (51.5±8.8 years old, range 20 to 82 years) and 3,842 women (50.3±8.7 years old, range 20 to 87 years). This study was approved by the ethics committees of the University of Tokyo, and written informed consent was obtained from each subject according to the Declaration of Helsinki.

### Diagnoses of the Four Acid-related Upper Gastrointestinal Disorders

Gastric ulcer (GU) and duodenal ulcer (DU) were diagnosed by endoscopy. In the present study, only active ulcers were considered as GU or DU respectively. Peptic ulcer (PU) was defined as the presence of GU and/or DU. Reflux esophagitis (RE) was also diagnosed by endoscopy, according to the modified Los Angeles (LA) classification [38]. Non-erosive reflux disease (NERD) was defined as the presence of heartburn and/or acid regurgitation among the subjects with no esophageal mucosal break [39]. To evaluate the symptoms of heartburn and acid regurgitation, two questions in the above-mentioned FSSG were used [37].

### Evaluation of Serum Anti-Helicobacter Pylori Antibody and Serum Pepsinogen Levels

Serum anti-*Helicobacter pylori* antibody was measured using a commercial EIA kit (E-plate “EIKEN” *H. pylori* antibody, EIKEN Chemical Co Ltd, Tokyo, Japan). According to the manufacture’s instruction, the antibody titer above 10 U/ml was considered as *HP*-positive. Serum pepsinogen I and II were measured using a commercial LAR kit (LZ test “EIKEN” pepsinogen I and pepsinogen II, EIKEN Chemical Co Ltd).

### Questionnaires

The Frequency Scale for the Symptoms of GERD (FSSG) is a widely used questionnaire for diagnosis of GERD and also for evaluating the effectiveness of digestive drug treatment [37]. Along with FSSG, a detailed questionnaire investigating symptoms related to the upper gastrointestinal disorders, the medical history, lifestyle factors, coffee consumption, etc., was given to all the participants. We analyzed answers for six questions as follows: i) “How often do you drink alcohol in a week?”; ii) “Do you have a habit of smoking?”; iii) “Have you ever undergone an eradication therapy for *Helicobacter pylori*?”; iv) “Do you have a history of gastric surgery?”; v) “Are you taking proton pump inhibitors (PPIs) or histamine H_2_-receptor antagonists (H_2_RAs)?”; and vi) “How much coffee do you drink?”. The answers for question i) were selected from five classifications (never, seldom, sometimes, often, and always), which were further categorized into two groups as nominal variables: rarely drinking group (never or seldom) and usually drinking group (sometimes, often, or always). The answers for question ii) were categorized into two groups as nominal variables: current or past habitual smoking (smoker group), and lifelong nonsmoking (nonsmoker group). The answer for iii), iv), and v) were “yes” or “no”. The answers for question vi) were categorized into three groups as ordinal variables: drinking less than a cup of coffee per day, 1–2 cups of coffee per day, and 3 or more cups of coffee per day.

### Meta-Analysis

The meta-analysis was conducted according to the PRISMA guidelines (Figure S1). Previous studies used in our meta-analysis were selected based on the inclusion criteria as follows: case-control or cohort design, registered in PubMED, CiNii (Scholarly and Academic Information Navigator) or Ichushi Web (NPO Japan Medical Abstracts Society) databases, statistically evaluating the association between coffee consumption and some ulcer disease (GU, DU, or PU), and describing the disease frequencies corresponding to all categories of coffee intake. The data sources were searched from September 2011 to September 2012. We excluded studies showing the results of significance but lacking the data on disease frequencies, because we cannot calculate the odds ratio in meta-analysis [7], [23], [40]–[42]. We adopted random effects meta-analysis method, because we assume that the analyzed datasets have a distribution with some central value and some degree of variability. All the results were presented graphically in forest plots, in which the diamonds at the bottom represent the pooled odds ratios of overall studies with the 95% confidence interval. In the forest plots, vertical lines (1) representing no effect were also demonstrated, which made us easy to grasp significance of odds ratios for all analyzed studies (shown as gray boxes) and overall pooled one (shown as a diamond). Major risks of bias in our meta-analyses were different designs for respective studies and a small number of eligible reports. We therefore performed a test for heterogeneity using a Cochran’s Q-statistics and I^2^ statistics.

### Statistical Analysis

The association of candidate background factors with the four major upper-gastrointestinal acid-related diseases was evaluated by univariate and multivariate analyses using the JMP® 9 program (SAS Institute Inc., Cary, NC, USA). After subjects with missing values were omitted, subjects with prior gastric surgery, taking PPIs and/or H_2_RAs, and having past history of *HP* eradication were further excluded from the study population, since such background factors might adversely affect accurate analysis. In the present study, we used eight factors as explanatory variables: age, body mass index (BMI), gender, drinking habit, smoking habit, *Helicobacter pylori* infection status, ratio of pepsinogen I/pepsinogen II (PG I/II ratio), and coffee consumption.

We categorized age into five groups to apply a univariate analysis: <40, 40–49, 50–59, 60–69, and ≥70. BMI and PG I/II ratio were respectively categorized into three groups: <18.5 (underweight), 18.5–24.9 (normal range), and ≥25.0 (overweight) for BMI; <2.0, 2.0–2.9, and ≥3.0 for PG I/II ratio. Based on the above-mentioned criteria, smoking, alcohol drinking, and *HP* infection status were divided into two groups: smoker and nonsmoker; drinking and rarely drinking; *HP*-positive and *HP*-negative.

Univariate analyses were done using Pearson's chi-square test, Student's t-test, and Welch's t-test to evaluate association between coffee consumption and other background factors. In addition, multiple logistic regression analysis was applied for evaluating the relationship between the above four esophago-gastro-duodenal diseases and eight background factors respectively. Specifically, we applied firth's penalized-likelihood method to deal with issues of separability, small event sizes, and bias of the parameter estimates for GU and DU. Age, BMI, and PG I/II ratio were evaluated as continuous variables, whereas smoking, alcohol drinking, *HP* infection status, and coffee consumption were analyzed as ordinal or nominal variables. A *p*-value of less than 0.05 was considered significant.

## Results

### Study Subjects

**Figure 1:**
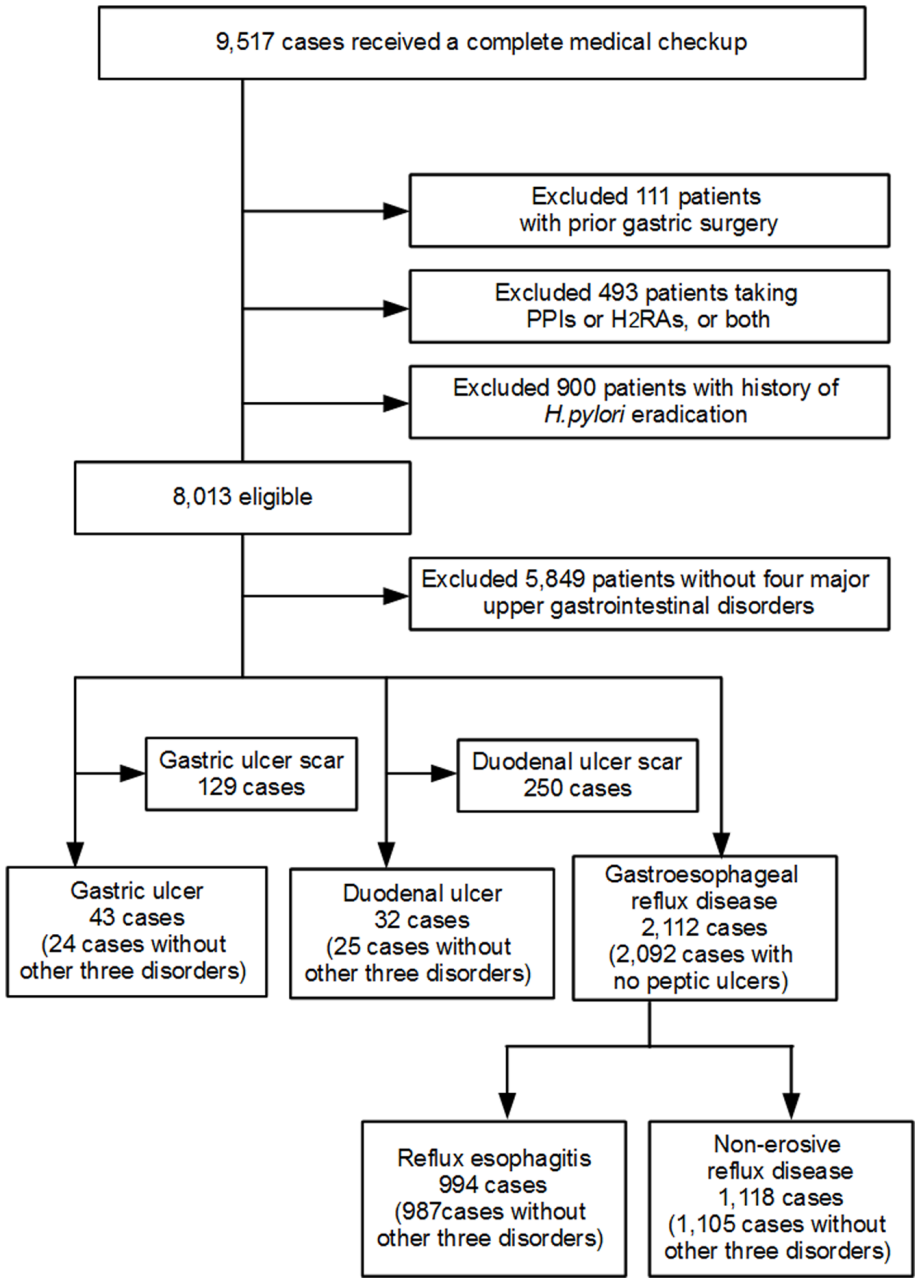
Study recruitment flowchart. Of the 9,517 healthy adults, we excluded subjects with prior gastric surgery (111), taking PPIs and/or H_2_RAs (493), and having history of *Helicobacter pylori* eradication (900). Among the eligible 8,013 subjects, numbers of subjects with GU, DU, RE, NERD, and other subjects free from the four major upper gastrointestinal disorders are shown.

**Figure 2:**
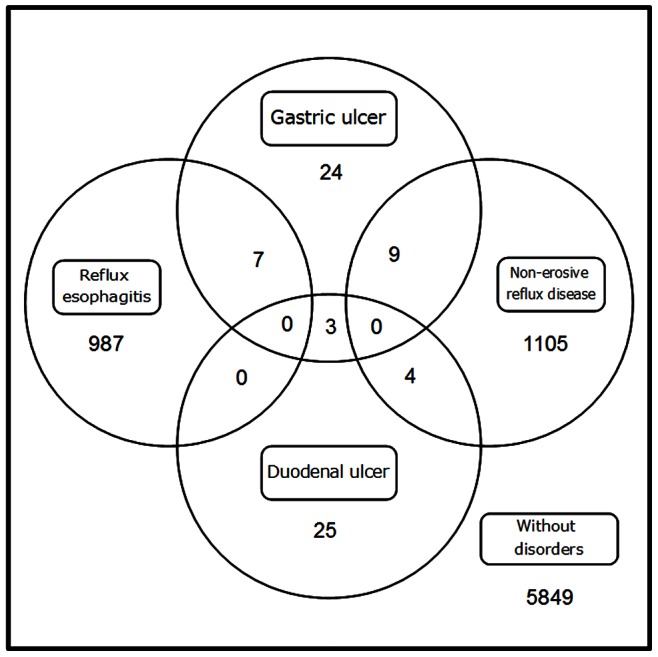
A venn diagram showing numbers of the four acid-related upper gastrointestinal disorders in our cohort.

Of the 9,517 subjects who attended this study, we excluded 1,504 subjects due to a history of gastric surgery (111), intake of PPI and/or H_2_RAs (493), and a history of *H. pylori* eradication (900). Among the eligible 8,013 study subjects (4,670 men and 3,343 women, 50.4±8.8 years old, range 20 to 84 years), 5,849 had neither PU nor GERD (Figure 1). Among the residual 2,164 subjects, 43 subjects (0.5%) had GU; 32 subjects (0.4%) had DU; 994 subjects (12.4%) had RE; and 1,118 subjects (14.0%) had NERD (Figure 1). Distribution of subjects with these four disorders is represented by venn diagram (Figure 2), in which one glance is enough to grasp the much higher prevalence of GERD (RE and NERD) in comparison with PU (GU and DU).

### Coffee Consumption and Seven Background Factors

Characteristics of our study population are shown in Table 1, which is categorized based on the presence of coffee intake. Between the coffee drinkers (one or more cups of coffee per day) and non-drinkers (less than a cup of coffee per day), age, BMI, PG I/II ratio, smoking, and alcohol drinking showed statistically significant difference, whereas gender and *HP* infection status did not (Table 1). From our results, coffee drinkers tend to be younger, smoke, drink alcohol, and present a higher level of PG I/II ratio.

**Table 1:** Characteristics of the study population and univariate analysis of risk factors for coffee.

	Drinker	Non-drinker		
	N = 5,451	N = 2,562		
	N (%)	N (%)	*p-value*	
**Age**				
** <40**	626 (67.6)	300 (32.4)	<0.001*	†
** 40–49**	1,937 (72.7)	727 (27.3)		
** 50–59**	2,265 (69.0)	1,017 (31.0)		
** 60–69**	583 (57.1)	438 (42.9)		
** 70≤**	40 (33.3)	80 (66.7)		
** Mean(±SD)**	49.8 (±8.2)	51.5 (±9.7)	<0.001*	‡
**Sex**				
** female**	3,194 (67.5)	1,476 (32.5)	0.405	†
** male**	2,257 (67.5)	1,086 (32.5)		
**BMI**				
** <18.5**	302 (63.7)	172 (36.3)	0.020*	†
** 18.5–24.9**	3,921 (68.9)	1,772 (31.1)		
** 25≤**	1,228 (66.6)	618 (33.4)		
** Mean(±SD)**	22.9 (±3.2)	23.1 (±3.5)	0.059	‡
**PG-I/PG-II**				
** <2**	312 (62.3)	189 (37.7)	0.003*	†
** 2–2.9**	588 (65.8)	306 (34.2)		
** 3≤**	4,553 (68.8)	2,065 (31.2)		
** Mean(±SD)**	5.4 (±2.0)	5.2 (±2.1)	0.007*	¶
**Smoking**				
** nonsmoker**	2,695 (64.4)	1,487 (35.6)	<0.001*	†
** former smoker**	1,567 (67.8)	744 (32.2)		
** current smoker**	1,189 (78.2)	331 (21.8)		
**Alcohol**				
** rarely drinking**	2,028 (65.2)	1,081 (34.8)	<0.001*	†
** usually drinking**	3,423 (69.8)	1,481 (30.2)		
***H. pylori***				
** positive**	1,681 (67.1)	823 (32.9)	0.247	†
** negative**	3,770 (68.4)	1,739 (31.6)		

* A *p-value* less than 0.05 was considered statistically significant.

† Pearson's chi-square test;

‡ Welch's t test;

¶ Student's t-test.

Prevalence of the four upper-gastrointestional disorders are next shown in Table 2, in which the study subjects were classified into three categories based on the coffee consumption per day. In our study cohort, almost 30% of study subjects have one or more acid-related upper gastrointestinal disorders. By the univariate analysis, we could detect no noticeable association between the degree of coffee consumption and all the four disorders (Table 2).

**Table 2:** The presence or absence of disorders with coffee consumption (in cups/day).

		without disorders		Gastric ulcer		Duodenal ulcer		Reflux esophagitis		Non-erosive reflux disease
Coffee consumption per day	No of subjects	N (%)	*p-value*	N (%)	*p-value*	N (%)	*p-value*	N (%)	*p-value*	N (%)
<1/day	2,562	1,848 (31.6)	0.229	14 (32.6)	0.093	12 (37.5)	0.360	339 (34.1)	0.174	358 (32.0)
1–2/day	2,978	2,206 (37.7)		10 (23.2)		8 (25.0)		345 (34.7)		414 (37.0)
≥3/day	2,473	1,795 (30.7)		19 (44.2)		12 (37.5)		310 (31.2)		346 (31.0)
Total	8,013	5,849		43		32		994		1,118

Include overlapping disorders of Gastric ulcer, Duodenal ulcer, Reflux esophagitis and Non-erosive reflux disease.

Cochran–Armitage test for trend.

### Peptic Ulcer

For gastric ulcer, we compared GU patients (n = 43) with GU-free subjects (n = 7,970). By multiple logistic regression analysis (Table 3), BMI, PG I/II ratio, smoking, and *HP* infection status showed a significant association with GU. Judging from the value of standardized coefficients (β), positively correlated factors of gastric ulcer in order of significance are *HP* infection (β = 0.746; OR = 18.55), current smoking (β = 0.275; OR = 3.57), higher BMI (β = 0.253; OR = 1.15), and higher PG I/II ratio (β = 0.248; OR = 1.24). Coffee consumption as well as age, sex, and alcohol drinking did not show significant association with GU.

**Table 3:** Summary of the estimate of Peptic Ulcer Diseases in multiple logistic regression analysis.

	Gastric ulcer (N = 8,013)			Duodenal ulcer (N = 8,013)		
	Standardized Coefficient	Odds Ratio (95% CI)	*p-value*	Standardized Coefficient	Odds Ratio (95% CI)	*p-value*
**Age**	0.173	1.04 (0.99–1.08)	0.066	−0.024	1.00 (0.95–1.04)	0.811
**Sex**						
** female**		reference			reference	
** male**	−0.031	0.89 (0.40–2.14)	0.780	0.152	1.75 (0.65–5.23)	0.252
**BMI**	0.253	1.15 (1.06–1.24)	<0.001*	−0.101	0.95 (0.83–1.06)	0.332
**PG-I/PG-II**	0.248	1.24 (1.01–1.50)	0.022*	0.420	1.45 (1.17–1.74)	<0.001*
**Smoking**						
** nonsmoker**		reference			reference	
** former smoker**	0.187	2.12 (0.89–5.31)	0.083	−0.053	0.81 (0.28–2.26)	0.669
** Smoker**	0.275	3.57 (1.49–8.98)	0.003*	0.184	2.35 (0.98–5.94)	0.048*
**Alcohol**						
** rarely drinking**		reference			reference	
** usually drinking**	−0.018	0.94 (0.48–1.90)	0.841	0.016	1.03 (0.79–1.37)	0.336
**Coffee**						
** <1/day**		reference			reference	
** 1–2/day**	−0.104	0.68 (0.29–1.52)	0.329	−0.166	0.54 (0.21–1.28)	0.145
** ≥3/day**	0.059	1.26 (0.62–2.61)	0.505	−0.059	0.79 (0.35–1.80)	0.557
***H. pylori***						
** negative**		reference			reference	
** positive**	0.746	18.55 (6.89–53.72)	<0.001*	0.924	37.23 (12.39–127.30)	<0.001*

* : A *p-value* less than 0.05 was considered statistically significant.

For duodenal ulcer, we compared DU subjects (n = 32) with DU-free subjects (n = 7,981). By multiple logistic regression analysis (Table 3), PG I/II ratio, smoking, and *HP* infection status showed a significant association with DU. Judging from the value of standardized coefficients (β), positively correlated factors of duodenal ulcer in order of significance are *HP* infection (β = 0.924; OR = 37.23), higher PG I/II ratio (β = 0.420; OR = 1.45), and current smoking (β = 0.184; OR = 2.35). Coffee consumption as well as age, sex, BMI, and alcohol drinking did not show significant association with DU.

### Gastroesophageal Reflux Disease (*GERD*)

For reflux esophagitis, the subjects with RE (n = 994) were compared with GERD-free subjects (n = 5,901). By multiple logistic regression analysis (Table 4), age, gender, BMI, PG I/II ratio, smoking, alcohol drinking, and *HP* infection showed a significant association with RE. Judging from the value of standardized coefficients (β), positively correlated factors of reflux esophagitis in order of significance are *HP* non-infection (β = 0.482; OR = 1/0.35 = 2.86), male gender (β = 0.426; OR = 2.37), higher BMI (β = 0.399; OR = 1.13), higher PG I/II ratio (β = 0.220; OR = 1.11), current smoking (β = 0.214; OR = 1.62), alcohol drinking (β = 0.143; OR = 1.34), and former smoking (β = 0.109; OR = 1.24). Among the examined variables, only coffee consumption did not show significant association with RE.

**Table 4:** Summary of the estimate of GERD syndrome in multiple logistic regression analysis.

	Reflux esophagitis (N = 6,895)			Non-erosive reflux disease (N = 7,019)		
	Standardized Coefficient	Odds Ratio (95% CI)	*p-value*	Standardized Coefficient	Odds Ratio (95% CI)	*p-value*
**Age**	0.159	1.02 (1.01–1.03)	<0.001*	−0.154	0.98 (0.97–0.99)	<0.001*
**Sex**						
** female**		reference			reference	
** male**	0.426	2.37 (1.95–2.90)	<0.001*	−0.125	0.78 (0.66–0.91)	0.002*
**BMI**	0.399	1.13 (1.11–1.15)	<0.001*	0.073	1.02 (1.00–1.04)	0.035*
**PG-I/PG-II**	0.220	1.11 (1.06–1.17)	<0.001*	−0.031	0.99 (0.94–1.03)	0.521
**Smoking**						
** nonsmoker**		reference			reference	
** former smoker**	0.109	1.24 (1.04–1.49)	0.019*	0.086	1.19 (0.63–1.32)	0.048*
** smoker**	0.214	1.62 (1.33–1.98)	<0.001*	0.139	1.36 (1.12–1.64)	0.002*
**Alcohol**						
** rarely drinking**		reference			reference	
** usually drinking**	0.143	1.34 (1.14–1.58)	<0.001*	0.059	1.13 (0.98–1.30)	0.093
**Coffee**						
** <1/day**		reference			reference	
** 1–2/day**	−0.062	0.88 (0.74–1.04)	0.133	−0.036	0.93 (0.79–1.08)	0.336
** ≥3/day**	−0.081	0.84 (0.70–1.01)	0.057	−0.032	0.93 (0.79–1.10)	0.408
***H. pylori***						
** Negative**		reference			reference	
** positive**	−0.482	0.35 (0.28–0.45)	<0.001*	0.065	1.15 (0.94–1.40)	0.158

* : A *p-value* less than 0.05 was considered statistically significant.

For non-erosive reflux disease (NERD), the subjects with NERD (n = 1,118) were compared with GERD-free subjects (n = 5,901). By multiple logistic regression analysis (Table 4), age, gender, BMI, and smoking showed a significant association with NERD. Judging from the value of standardized coefficients (β), positively correlated factors of NERD in order of significance are younger age (β = 0.154; OR = 1/0.98 = 1.02), current smoking (β = 0.139; OR = 1.36), female gender (β = 0.125; OR = 1/0.78 = 1.28), former smoking (β = 0.086; OR = 1.19), and higher BMI (β = 0.073; OR = 1.02). Coffee consumption as well as PG I/II ratio, alcohol drinking, and *HP* infection status did not show significant association with NERD.

### Meta-Analysis

**Figure 3:**
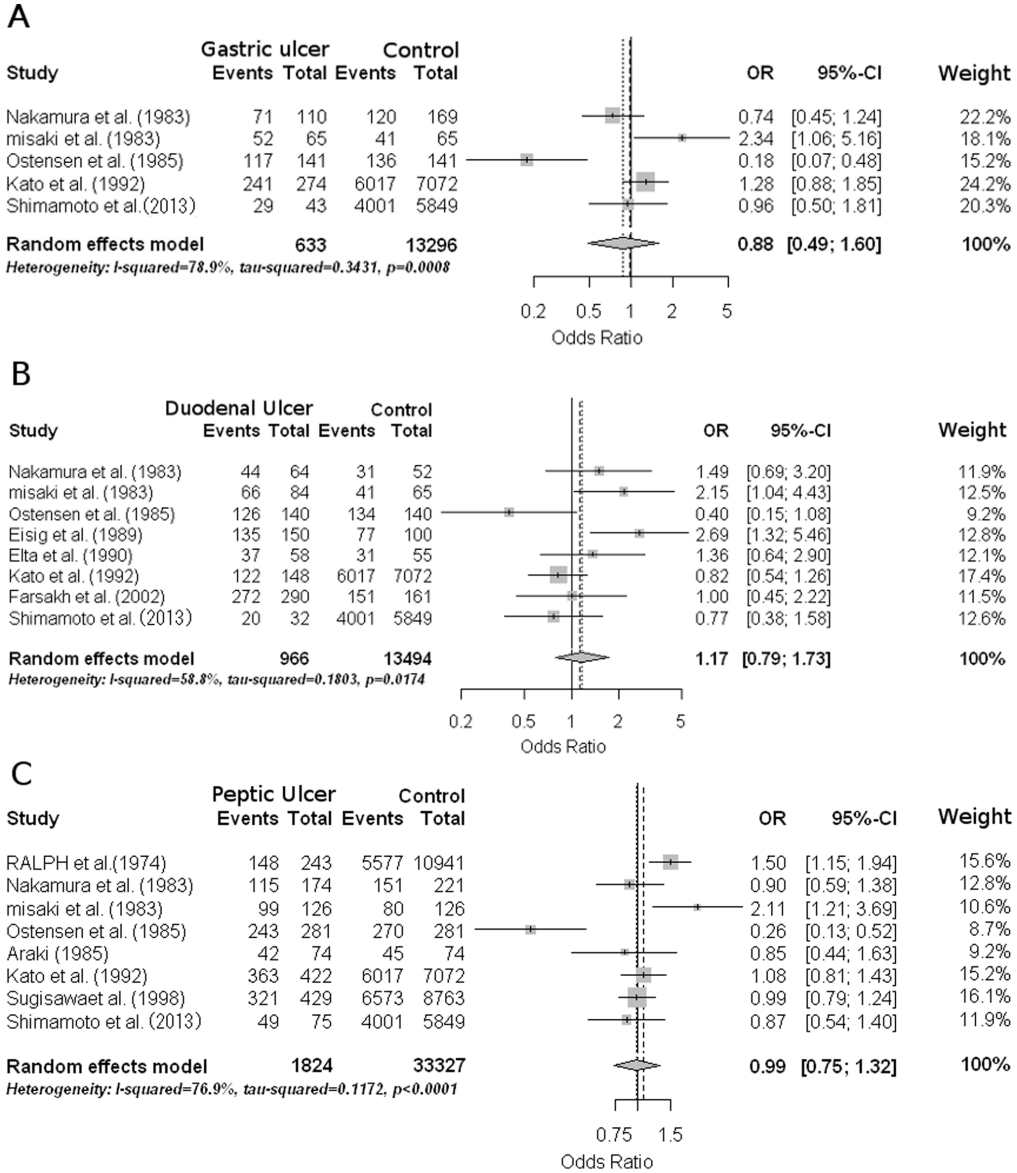
Forest plots of the odds ratios and 95% confidential intervals for upper gastrointestinal peptic ulcer. Forest plots of the odds ratios and 95% confidential intervals for gastric ulcer (A), duodenal ulcer (B), and peptic ulcer (C) relating coffee intake. The gray box represents the odds ratio estimates in each study, and the horizontal line indicates the 95% confidential intervals for each study. Diamonds at the bottom represent the pooled odds ratio estimates. Weights are from random effect meta-analysis.

To get an overview of the accumulated reports concerning association between coffee consumption and the four upper gastrointestinal disorders, we performed meta-analyses using the random effects model. For peptic ulcer diseases, we found six case-control and four cohort studies fulfilling the inclusion criteria for meta-analysis (Table S1). We have found other five studies evaluating the association of coffee with peptic ulcer diseases (Table S2), which did not meet the criteria and could not be used in our meta-analysis. A total of 10 papers met the inclusion criteria and were included in the meta-analysis, together with our present study (Figure 3). For GERD (RE and NERD), we could not perform meta-analysis, because a number of studies meeting the criteria was too small.

Meta-analysis was executed using a random effects model, because a test of heterogeneity was statistically significant. As shown in Figure 3A, the meta-analysis of two case-control and three cohort studies showed no significant association between coffee consumption and GU (pooled odds ratio, 0.88; 95% CI, 0.49 to 1.60; *p* for heterogeneity, 0.0008; I^2^, 78.9%). The meta-analysis of five case-control and three cohort studies also detected no significant association between coffee consumption and DU (Figure 3B; pooled odds ratio 1.17; 95% CI, 0.79 to 1.73; *p* for heterogeneity, 0.0174; I^2^, 58.8%). For PU, we analyzed three case-control and five cohort studies (Figure 3C), which again denied significant association with coffee consumption (pooled odds ratio 0.99; 95% CI, 0.75 to 1.32; *p* for heterogeneity, <0.0001; I^2^, 76.9%). To sum up, the meta-analyses could not detect a significant association of coffee consumption with the upper gastrointestinal ulcer diseases.

Publication bias of each meta-analysis was further assessed by a funnel plot and funnel plot regression, in which *p*-values less than 0.1 were considered statistically significant. In all meta-analyses we performed, significance tests of the asymmetry were not significant (GU: *p* = 0.582, DU: *p* = 0.146, PU: *p* = 0.396). We thence concluded that publication bias can be considered as nonsignificant.

## Discussion

All the four upper gastrointestinal disorders examined in the present study have been considered as acid-related diseases [3]. Therefore, it is easy to conceive that coffee containing caffeine stimulates the gastric acid production [4], [43]–[45], and consequentially increases the risk of these disorders. Especially for PU, it has been repeatedly reported that the coffee is a risk factor for both gastric and duodenal ulcer [9], [16]. However, multivariate analysis of the healthy subjects (Table 3) could not detect significant association between coffee intake and upper gastroduodenal ulcer diseases. The meta-analysis including our present study was further conducted, which denied the meaningful association between them (Figure 3). We speculated that some preventive effects of coffee intake might outweigh the risks of increased gastric acid secretion: relaxing effect, antioxidant effect, phytochemical effect, and so on [46]–[48].

For GERD, we also could not detect a significant association between coffee intake and the incidence of GERD (both RE and NERD), although some past study have reported that coffee intake may predispose to GERD syndrome [19]. Besides the stimulating effect upon gastric acrid production, it was also reported that coffee intake relaxes the lower esophageal sphincter [49], which might cause the chronic gastric acid reflux. Excessive secretion of gastric acid can damage not only the gastroduodenal but also esophageal mucosa, but our multivariate analysis of the healthy subjects (Table 3) did not detect significant association between coffee consumption and GERD (both RE and NERD). At present, epidemiological studies concerning coffee intake and GERD have been very few. Many studies like ours should be accumulated in the future, which will make it possible to perform the reliable meta-analysis.

One of limitations of our present study was of course a cross-sectional design, which should be precisely validated in the future prospective study. We are following the present large-scale cohort to validate our present conclusion in the upcoming trial. Another limitation of our study was lacking more detailed information of coffee, such as kinds of coffee beans, use of milk or sugar, regular coffee or not, the time of coffee drinking, etc. These minute data concerning coffee intake will be added to the future study, which will make our next research more accurate and polished to verify our present conclusion.

## Supporting Information

Figure S1 **Flow diagram of the meta-analysis literature search results.**
(DOC)

Table S1 **Summary characteristics of cohort or case-control studies were included from the meta-analysis that compare the relationship of coffee and peptic ulcer.**
(XLS)

Table S2 **Summary characteristics of cohort or case-control studies were excluded from the meta-analysis that compare the association of coffee and peptic ulcer.**
(XLS)

Document S1 **References used in Table S1 & S2.**
(DOC)
