# Supplementary material for: No Association of Coffee Consumption with Gastric Ulcer, Duodenal Ulcer, Reflux Esophagitis, and Non-Erosive Reflux Disease: A Cross-Sectional Study of 8,013 Healthy Subjects in Japan
Source: PLoS One. 2013 Jun 12;8(6):e65996. doi: 10.1371/journal.pone.0065996 (PMC3680393; doi:10.1371/journal.pone.0065996)
Supplement: Figure S1 — Flow diagram of the meta-analysis literature search results. (DOC) [file pone.0065996.s001.doc]

PRISMA Flow Diagram

**Screening**

**Included**

**Eligibility**

**Identification**

Records excluded,

Not investigate the relationship between coffee and peptic ulcer
(n = 32 )

Records identified through database searching
(n = 47 )

Additional records identified through other sources
(n = 0 )

Records after duplicates removed
(n = 0 )

Records screened
(n = 15 )

Full-text articles assessed for eligibility
(n = 10 )

Full-text articles excluded,

Cannot compute odds ratio for the absence of count data
(n = 5 )

Studies included in qualitative synthesis
(n = 10 )

Studies included in quantitative synthesis (meta-analysis)
(n = 10 )
