# Supplementary material for: No Association of Coffee Consumption with Gastric Ulcer, Duodenal Ulcer, Reflux Esophagitis, and Non-Erosive Reflux Disease: A Cross-Sectional Study of 8,013 Healthy Subjects in Japan
Source: PLoS One. 2013 Jun 12;8(6):e65996. doi: 10.1371/journal.pone.0065996 (PMC3680393; doi:10.1371/journal.pone.0065996)
Supplement: Document S1 — References used in Table S1 & S2. (DOC) [file pone.0065996.s004.doc]

**Supplemental document**

**References used in Table S1 and Table S2**

1. Nakamura T, Kamakami T, Ohkuni A, et al. Effects of smoking, alcohol and coffee drinking on the course of peptic ulcer. *Nihon Shokakibyo Gakkai Zasshi* 1983; **80**: 2493-503.

2. Misaki F, Hayashi K, Watanabe Y, et al. An epidemiological study on risk factors in peptic ulcer. *Nihon Shokakibyo Gakkai Zasshi* 1983; **80**: 2504-11.

3. Ostensen H, Gudmundsen TE, Ostensen M, et al. Smoking, alcohol, coffee, and familial factors: any associations with peptic ulcer disease? A clinically and radiologically prospective study*.* *Scand J Gastroenterol* 1985; **20**: 1227-35.

4. Eisig JN, et al. Coffee drinking in patients with duodenal ulcer and a control population*.* *Scand J Gastroenterol* 1989; **24**: 796-8.

5. Elta GH, EM Behler, TJ Colturi. Comparison of coffee intake and coffee-induced symptoms in patients with duodenal ulcer, nonulcer dyspepsia, and normal controls*.* *Am J Gastroenterol* 1990; **85**: 1339-42.

6. Kato I, Abraham M, Nomura Y, et al. A prospective study of gastric and duodenal ulcer and its relation to smoking, alcohol, and diet*.* *Am J Epidemiol* 1992; **135**: 521-30.

7. Abu Farsakh NA. Risk factors for duodenal ulcer disease*.* *Saudi Med J* 2002; **23**: 168-72.

8. Paffenbarger RS Jr, AL Wing, RT Hyde. Chronic disease in former college students; 13. Early precursors of peptic ulcer. *Am J Epidemiol* 1974; **100**: 307-15.

9. Araki S. The factors affecting gastric and duodenal ulcers in Japanese factory workers. A case-control study*.* *Sangyo Igaku* 1985; **27**: 242-7.

10. Atsuko S, Tetsunojo U. Onset of Peptic Ulcer and Its Relation to Work-Related Factors and Life Events : A Prospective Study. *Journal of occupational health* 1998; **40**: 22-31.

11. Friedman GD, AB Siegelaub, CC Seltzer. Cigarettes, alcohol, coffee and peptic ulcer*.* *N Engl J Med* 1974; **290**: 469-73.

12. Nechige R, et al. Back ground Factors of Peptic Ulcer*.* *Yamaguchi medical journal* 1981; **30**: 455-462.

13. Watanabe Y, Kurata JH, Kawamoto K, et al. Epidemiological study of peptic ulcer disease among Japanese and Koreans in Japan*.* *J Clin Gastroenterol* 1992; **15**: 68-74.

14. Aldoori WH, Giovannucci EL, Stampfer MJ, et al. A prospective study of alcohol, smoking, caffeine, and the risk of duodenal ulcer in men*.* *Epidemiology* 1997; **8**: 420-4.

15. Rosenstock S, , Jørgensen T, Bonnevie O, et al. Risk factors for peptic ulcer disease: a population based prospective cohort study comprising 2416 Danish adults*.* *Gut* 2003; **52**: 186-93.
